# Tough versus soft regulations to promote generic medications in Italy

**DOI:** 10.1007/s10198-025-01826-y

**Published:** 2025-09-02

**Authors:** Aarushi Dhingra, Gianluca Fiorentini, Ayman Fouda, Naomi Moy

**Affiliations:** 1 Department of Economics, University of Bologna, Bologna, Italy; 2 MCI Management Center Innsbruck, Innsbruck, Austria; 3 Department of Gastroenterology and Hepatology, Princess Alexandra Hospital, Brisbane, Australia

**Keywords:** Prescription decision, Generic drugs, Medication-naivety, Multi-agent problem, Chronic kidney disease, I18

## Abstract

This paper investigates the effects of regulations aiming to optimise the multi-agency relationships, with a focus on the prescription choice between generic versus branded medications. In 2012, Italian legislators introduced two laws targeting general practitioners prescription behaviour, a soft (recommendations) law followed by a hard (mandatory) law to promote generic medication. We implement a regression discontinuity in time framework and an event study to quarterly administrative data for individuals with chronic kidney disease linked to data from their general practitioners in Emilia-Romagna, Italy. The results indicate that the policies were effective, but had modest effects on increasing the prescription of generic medications. Hard laws seem to have played a key role in driving this change. Heterogeneity checks provide evidence that less competition among GPs and interaction with specialists increased generic medication prescriptions.

## Introduction

Prescription regulations are frequently used to try to influence the prescribing behaviour of medical professionals in a setting where physicians act as agents of multiple principals, patients and regulators. On the one hand, patients want their physician to act as their agent, in providing information and medical care services. On the other hand, the planners want the physician to act in their best interest, which may or may not align with those of the patients leading to varying physician behaviour [6, 11, 46]. Since prescribing behaviour is a key intervention area for optimising health expenditure, influencing the tripartite interaction between agents is vital for improving the appropriateness of care and ensuring the sustainability of health systems [25, 41]

In its essence, this paper investigates the effects of two law changes, that aim to increase the prescription of generic drugs. These medications contain the same active ingredients as branded drugs but differ only in appearance and packaging, and are sold at lower prices once patents expire [14].^1^ We study the effect of two law changes targeting the supply side of medications in Italy. The first law was a soft law (recommendations) implemented in January 2012 while the second law (mandatory norms) was implemented in August 2012 targetting medication naïve patients. Both laws aimed to change physician prescribing behaviour to promote generic medications. We use individual – physician linked sources of quarterly administrative data, from 2009 to 2016 for a sample of patients with chronic kidney disease (CKD).^2^

Since the laws aim to target different populations, we implement a regression discontinuity in time (RDiT) and a dynamic difference in difference, labelled as an event study, to estimate the effect of these closely implemented laws. For the event study we conduct two separate analyses 1) by identifying the specific effects of the soft and the hard law, using two separate quarterly analyses; 2) by ’bundling’ them as one law and studying its effect on yearly use of generic medications. The analysis is conducted from an individual perspective rather than the perspective of the GPs despite the availability of data for GPs. This is because we have access to individual level data for patients with CKD and their corresponding GPs, meaning that a GP-focused analysis might provide a biased representation of GP behaviour, as it would only reflect their interactions with CKD patients rather than their broader patient pool.

Research on the relationship between pharmaceuticals and rising health expenditure has been growing for decades. In several middle-to-high income economies, pharmaceuticals have been identified as one of the main drivers of rising health expenditure [4, 13, 44]. According to the European Commission many patients do not benefit from pharmaceuticals innovations, because of the high costs or availability [13]. The affordability of medicines has implications for both public and household finances, for all member States in Europe and around the globe [13]. As highlighted in the Pharmaceutical Strategy for Europe, the European Commission is considering target policies that support and provide greater access to generic medications.

Given that most healthcare systems in Europe subsidise or fully cover the cost of essential medications, our results generalise to contexts where rising pharmaceutical costs and budget pressures challenge sustainable access. Our results are timely and relevant in the context of rising multi-morbidity and related polypharmacy or hyper-polypharmacy driven by ageing populations globally [20] and leading to a significant increase in the prescription of medications for which there are both branded and generic versions. Additionally, while our analysis centres on CKD patients, the results are generalisable to other chronic conditions. Since these policies target GP’s prescribing practices, rather than being disease-specific, a GP’s decision to prescribe generics should not vary based on the condition being treated. Moreover, according to the clinical literature more than 90% of the CKD patients are multi-morbid (especially diabetes and cardio-vascular conditions), thereby requiring multiple medications [21].

Previous studies, primarily from the United States, have evaluated the effectiveness of different state-level generic substitution laws aimed at increasing generic drug uptake. Evidence suggests that laws granting pharmacists greater discretion, such as presumed consent policies can modestly reduce the likelihood of purchasing branded name [30, 35, 43], reduced government spending on prescriptions [17]. Shrank et al. [39] argue that permissive policies with minimal enforcement lead to lower substitution rates and policymakers need to consider patients’ autonomy. In the European context, Andersson et al. [2] provide evidence from Sweden showing that mandatory substitution laws significantly reduced both patient and societal pharmaceutical expenditures.

Several factors, on both the supply and demand side have been identified to influence the prescribing behaviour [40, 42]. On the supply side, competition plays a role in affecting both the volume and the type of medication. Schaumans [38] found that under competitive pressure, GPs tend to over-prescribe medications to their patients to signal a higher degree of care and hence, to retain them within their practices. This also helps them gain or preserve the market share of patients and avoid economic punishment. Payment incentives may also influence the prescribing behaviour [18]. Specifically, physicians whose incomes depend on the number of patients are less likely to promote generic medication due to the high opportunity cost of educating the patient about the medication, leading to fewer numbers of patient visits. Whereas, if physicians are allowed to dispense medications, they are more inclined to prescribe and promote generic medications as their income is associated with increases in generic pharmaceutical consumption [7, 33]. Analogously, if dispensing branded medications is more profitable, physicians are shown to promote those regardless of the financial burden incurred by the patient [23]. The type of physician and the patient history of hospitalisation have also been proven to have an effect on the prescription choice. In particular, outpatient physicians are less likely to prescribe generic medications to patients with a history of previous hospital visits [32].

On the demand side, the patients’ perception of the efficacy of generic medications may limit their diffusion [22, 40]. Habit, inertia, and risk aversion are also factors that favour the demand for branded medications [3, 25, 31]. Saposnik et al. [37] show that in a setting where the price difference between branded and generic alternatives is marginal, a higher risk propensity is associated with higher diffusion of generic medications. Income and prices also play a role in purchasing decisions, but the results are not conclusive. In settings where patients face co-payments for pharmaceuticals, generic medications are marginally preferred [10]. Similarly, in health systems with deductibles, patients with higher deductible levels are more likely to demand generic medications [33]. In contrast, the low price of generic medications does not seem to influence patients’ demand or to incentivise the switch from branded to generic [47]. Finally, individual characteristics such as age and ethnicity are also associated with the decision to switch from branded to generic medication [34].

Existing studies largely focus on the U.S. with no study addressing these particular laws in the Italian healthcare system, which operates under a universal healthcare system. No prior research has specifically examined the impact of generic promotion policies among physicians for medication naïve patients who may be more amenable to such substitution due to the absence of established brand preferences. Moreover, the role of GPs and how varying levels of local competition among providers influence prescribing behaviour remain under explored. Therefore, our contributions are fourfold. We address the gap in the literature by examining the effects of nationally implemented laws that target the physicians in assessing the impact on prescriptions, especially among medication naïve patients. Second, we attempt to disentangle the effects of two laws enacted in close temporal proximity, but targeting different population groups. Third, we contribute to the literature by analysing how two different laws affected generic prescriptions specifically for patients with or without previous interactions with the hospital specialists, as well as with GPs working in areas with different competitive pressures. Fourth contribution is to explore how the socioeconomic status (SES) of patients affects physicians’ prescribing behaviour.

The paper proceeds as follows: Section Institutional setting provides the institutional settings in the Emilia-Romagna region with respect to the guidelines of medication prescription and the regulation of physician practice. Section Data describes the data. Section Empirical methods describes the methods. Section Results presents the results. Section Robustness checks provides the robustness checks, Section Heterogenous effects the heterogeneity checks and Section Discussion and conclusion concludes.

## Institutional setting

The Italian national health authorities implemented several regulations aiming to promote the prescription of generic medications in the last two decades. The national Law 405/2001 introduced the requirement for pharmacists to inform patients about the availability of generic drugs if the physician had not vetoed them in 2001 [26]. In July 2010, the 2001 law components were preserved and patients were provided with clearer information about the price differential between generic and branded medications [28]. In January 2012, another piece of legislation was enacted to promote the use of generic drugs in the interaction between physicians and patients [29]. The latter constitutes the first law directly targeting the prescribing behaviour of physicians by making it mandatory for the physician to inform all patients that there are generic alternatives for the medication prescribed (hereafter labelled as ’soft law’). In August 2012, a second law targeting physicians’ behaviour was enacted (Law 135/2012) as a response to what was felt to be the lack of compliance with the soft law. This second law required physicians to prescribe a generic version of the medication and indicate only the active ingredient for *naïve patients*, i.e., patients without any previous medical experience with the drug or individuals who had previous experience with medications followed by a long washout period^3^ [5], without specifying commercial names in the prescription (hereafter labelled as the ’hard law’).

The two laws can be described as stepwise developments to promote generic medications through different degrees of law enforcement. The relatively short time span between the two measures, together with the lack of sanctions or systematic monitoring on the results of the soft law are additional signs that seem to indicate that most professionals regarded it as something similar to a clinical practice guideline.

In Emilia-Romagna, GPs are regulated by national and regional regulations. Two such important regulations are 1) the GP payment scheme(s) and 2) the number of patients per GP. For the former, capitation^4^ payment per patient is used to reimburse GPs. For the latter, the regulation stipulates a maximum capacity for GPs of 1, 500 patients except in specific circumstances [15]. These regulations were set in the national agreement between the National Health Service (NHS) and the Unions of the GPs in 2006 and have not changed thereafter. Such regulations contribute to creating a regulated competitive environment. On one hand, GPs are incentivised to add patients to their list up to approximately 1,500 patients to increase their income. This can be a challenging objective for those who operate in competitive geographic areas characterised by a high density of GPs. On the other hand, after passing the threshold, the incentive to add more patients is weakened as there is the risk that additional patients will not be allowed to opt for their preferred GP. Prescribed medications are supplied by pharmacies, with costs borne by the consumer. Medicines that are reimbursed by the NHS are either free of charge, or require payment of a fee (‘ticket’) determined by national and regional regulations.

## Data

We use data on health service consumption of CKD patients in the Emilia-Romagna Region of Italy. This anonymised dataset provides the quarterly health consumption of CKD patients between 2009 and 2016 and contains information on patients’ individual characteristics, hospitalisations, ambulatory services, laboratory tests, and pharmaceutical prescriptions. The data was collected every quarter from 44,686 individuals. Depending on the empirical method used, the analysis is conducted using 17 to a maximum of 32 quarters (8 years, 4 quarters per year). We exclude 15 paediatric patients (under 18 years old) in the first year of data collection, and an additional 1,430, as they did not consume any form of medication for the entire period nor were residents of Emilia-Romagna. The final dataset is an unbalanced panel (due to death or relocation to other Regions), including 42,954 individuals.

This dataset is merged with another dataset that includes Emilia-Romagna’s prescriptions^5^ of the ATC category of Hypertension drugs. This contains different types of licensed hypertension medications in the region for the period studied ($n = 2,094$) and differentiates medications by their type, generic or branded. Relevant individual patient-related characteristics and linked GP characteristics are included in the analysis. For the patients we include age, gender, health status, co-payment contributions, an indicator if they switched their GP and if they had visited a medical specialist in the past. We also include an indicator if the individual is medication naïve, which takes the value of 1 in the quarter of the first prescribed hypertension medications and 0 otherwise. Individual GP characteristics are also controlled for, such as age, gender, number of patients, working in a group practice, and GP density in the area of residence.

A summary of the individual patient characteristics is described in Table 1. On average, individuals are 78 years and the majority of patients are males. The average score for the Charlson Co-morbidity Index is 1.6 diseases. Moreover, naïve patients constitute 15% of the total number of observations and very few patients approximately 2% have switched their GPs. Regarding the involvement of other practitioners, in approximately 75% of the observations, specialists were either previously or simultaneously involved in managing patients.

For the GPs, the average age is 59 years and 26% are female. The average number of patients per GP is approximately 1,454 patients. Approximately 90% of the GPs are affiliated to GP group practices. The average number of GPs per district is 6.8/10, 000 population. The majority of GPs (53%) work in areas that are above the mean average of the GP density.

**Table 1:** Summary Statistics of the Patient and GP Characteristics

Variable	Mean	SD	Min	Max
*Patient Characteristics*				
Age	78.14	11.40	18	107
Female	0.411	0.492	0	1
Charlson Index	1.60	1.65	0	11
Chronic Kidney Disease Stage				
No CKD	0.410	0.49	0	1
Stage 1	0.037	0.19	0	1
Stage 2	0.13	0.34	0	1
Stage 3	0.16	0.37	0	1
Stage 4	0.06	0.23	0	1
Stage 5	0.02	0.14	0	1
End Stage	0.05	0.22	0	1
Unspecified	0.11	0.31	0	1
Co-payment(%)	9.29	9.97	0	100
Medicine Naïve	0.15	0.36	0	1
Specialist visit	0.75	0.44	0	1
GP Switching	0.02	0.13	0	1
Region				
Bologna	0.23	0.42	0	1
Cesena	0.06	0.24	0	1
Ferrara	0.09	0.29	0	1
Forlì	0.05	0.22	0	1
Imola	0.02	0.15	0	1
Modena	0.15	0.36	0	1
Parma	0.08	0.27	0	1
Piacenza	0.06	0.24	0	1
Ravenna	0.10	0.30	0	1
Reggio-Emilia	0.10	0.30	0	1
Rimini	0.06	0.24	0	1
*GP Characteristics*				
GP Age	58.85	4.88	34	70
GP Female	0.26	0.44	0	1
# patients (Yearly)	1454	305.80	1	2170
GP Density	6.86	0.97	3.46	8.821
GP high density	0.53	0.50	0	1
Observations	805,621			

Notes: The tables present the descriptive statistics for the explained and explanatory variables in the analysis. Mean represents the average of the variable across the quarter based on the sample and SD is the standard deviation. Min and Max are the minimum and maximum values the variables attain

## Empirical methods

To answer the research questions, we implement two different econometric approaches, RDiT and event study. RDiT is implemented to investigate the effect of the soft law that targeted all patients, whilst the event study is implemented to investigate the effect of the bundle law and hard law for medication naïve patients. Since the two laws were implemented in close temporal proximity, it is challenging to clearly separate their effects. However, we conduct distinct analyses for each in order to disentangle their individual impacts.

### Regression discontinuity in time

Since the soft law targeted all patients and all physicians, it is not possible to identify a treatment and control group at the patient level. As a result, we use an RDiT to estimate the local average treatment effect (LATE) to compare the treatment effects on a specific period, which is immediately before and after the enactment of the law. The following RDiT design is estimated to study the effect of the soft law:

1 $$\begin{aligned} y_{it} = \alpha + \beta PolicyQ_{t} + g(t) + \delta X_{it} + \epsilon _{it} \end{aligned}$$

Where $y_{it}$ takes the value 1 if generic medication was prescribed in that quarter and, 0 otherwise, see Table 2, $\beta$ corresponds to the magnitude of the discontinuity in prescribing behaviour at the policy date. *PolicyQ* represents the implementation of the soft law in January 2012 or Quarter 13 (Q13). Intuitively, the function *g*(*t*) controls for unobserved factors that evolved smoothly over time and are unrelated to the policy changes, $X_{it}$ are the control variables, for patients and GPs, described in the data section. $\epsilon _{it}$ is the error term.

RDiT approach is feasible in our setting as the assumptions for the same are met. The soft law allows the identification of a clear cut-off point on the running variable, which is January 2012. This is divided by quarters: 2 years (8 quarters) before and after the cut-off point. Previous literature requires observations far from the temporal threshold. Identification in cross-sectional regression discounting design rests upon a conditional expectation as one approaches the threshold, thus relying on a mass of cross-sectional units just above and below the threshold. However, in the RDiT framework, there is little or no cross-sectional identifying variation, therefore, the sample is too small for estimation as the bandwidth narrows around the threshold. As a result, many RDiT settings expand time bounds to obtain sufficient power, relying on observations away from the threshold, and we follow in the same footsteps. The estimation is conducted with a local linear RDiT estimator, triangular kernel function, minimised mean squared error of the RDiT estimator [9]. The standard errors are clustered at the GP level to account for intra-cluster correlation, GP’s prescribing behaviour for one of their patient is likely to be correlated with that for another patient. Additionally, such correlations is assumed to be contained within a GP’s practices and are independent across GPs.

**Table 2:** Dependent and Independent Variables

Variable	Type	Values	Mean	SD
Dependent Variables				
Generic Medication Indicator	Dummy	$= 1$ if generic medication, $= 0$ if branded	0.462	0.499
Explanatory Variables				
Medicine Naïve	Dummy	Naïve patients are the treatment group $= 1$ in the quarter of first and following prescribed hypertension medications, Naïve $= 0$ in all quarter for the control group i.e, Non-naïve patients.	0.149	0.356
Quarter Number	Categorical	Categorical variable denoting the quarters of observations. $= 1$ if Quarter 1 (January-March 2009); $= 2$ if Quarter 2 (April-June 2009); etc	14.20	8.237

Notes: The number of observation is equal to 805,621

### Event study to estimate the yearly effects

As the two laws were implemented over a short time span, it is challenging to clearly estimate their effects on medication naïve patients. We first estimate the effects on the prescription of generic medication, bundling the soft and the hard law using yearly data for medication naïve patients. In an attempt to disentangle each laws’ individual effect, we conduct separate analyses leveraging on quarterly data. In both cases, we conduct an event study design to examine the dynamic effects.

The relatively long panel allows for an event study in which the causal effects of the treatment can be observed over multiple time periods. We implement an event study to observe the effects of the laws together in each year following [8]. The event study allows us to investigate the existence of pre and parallel trends, before the laws were implemented. We focus on naïve patients, who did not consume medication in the first period of observation nor before the enactment of the law and received medication after the enactment of the laws. The following model is estimated:

2 $$\begin{aligned}& (Y_{it}) = \underbrace{{\underset{k\ne -1}{\overset{k=-1}{\underset{k=-3}{\sum }}}} \alpha _k D_{i} 1 (t-t_{i}^* = k )}_\text {Pre-treatment-effect} +\\&\underbrace{\sum _{k=0}^{k=4}\beta _k D_{i} 1 (t-t_{i}^* = k )}_\text {Post-treatment-effect} + \nu X_{it} + \gamma Z_{it} + c_{i} + \varphi _{t} + \epsilon _{it} \end{aligned}$$

On the left-hand side, $Y_{it}$, represents the average generic medications prescribed in a year, *t*. $t_{i}^*$ refers to the year in which the policies were implemented i.e. 2012 and *t* represents the year in which the medication is taken for an individual *i*. $1(t-t_{i}^*= k)$ takes a value of 1 when the year is $-3\ldots ,-1,\ldots 4$ relative to the year of policy intervention (year 2012), $t_{i}^*$. That is, using the dataset, an observation window of 3 years pre-policy change and 4 years post-policy change is constructed.

It is common practice in event study frameworks to set $k= -1$ (or year 2011) as the reference point, we follow in this tradition for this analysis. The main coefficients of interest are $\alpha _k$ and $\beta _k$ and that are deemed to be causal effects of the polices on generic medication prescription, where $\alpha _k$ reflects the pre-policy effects and $\beta _k$ post-policy effects on prescriptions. $X_{it}$ and $Z_{it}$ are the time-varying covariates for the individuals and the GPs, respectively. $c_i$ captures the individual fixed effects (FE) and $\varphi _t$ captures the calendar year effects. $\epsilon _{it}$ are the usual error terms. The standard errors are clustered at the individual level, since medication naïve is individual specific, and adjusted for heteroskedasticity.

### Event study to estimate the effects of the two laws separately

We try to disentangle the effects of the two laws. We now leverage the quarterly data, allowing us to conduct two separate event studies in which the causal effects for the soft and the hard law can be estimated for medication naïve patients. To estimate the effects of the soft law, the following model, presented in Eq. 3, is implemented. $Y_{it}$ take the value 1 if generic medication was prescribed in that quarter and, 0 otherwise. $t_{i}^*$ refers to the quarter of policy implementation i.e., January 2012, *t* the quarter in which the medication is taken for the individual *i*. $1(t-t_{i}^*= k)$ takes a value of 1 when the quarter is $-11,\ldots ,-1,\ldots 19$ to estimate the effect of the soft law, relative to one quarter before the policy was implemented, $t_{i}^*$. That is, using the dataset, an observation window of 11 quarters pre-policy change and 19 quarters post-policy change is constructed. One needs to be cautious when interpreting the results of this design, as 2 quarters after the implementation of the soft law, the hard law was implemented.

3 $$\begin{aligned}& (Y_{it}) = \underbrace{{\underset{k\ne -1}{\overset{k=0}{\underset{k=-11}{\sum }}}} \alpha _k D_{i} 1 (t-t_{i}^* = k )}_\text {Pre-treatment-effect} +\\&\underbrace{\sum _{k=1}^{k=19}\beta _k D_{i} 1 (t-t_{i}^* = k )}_\text {Post-treatment-effect} + \nu X_{it} + \gamma Z_{it} + c_{i} + \varphi _{t} + \epsilon _{it} \end{aligned}$$

Next, to estimate the results for the hard law the following model is implemented:

4 $$\begin{aligned}& (Y_{it}) = \underbrace{\underset{k\ne 0}{\overset{k=0}{\underset{k=-13}{\sum }}} \alpha _k D_{i} 1 (t-t_{i}^* = k )}_\text {Pre-treatment-effect} +\\&\underbrace{\sum _{k=1}^{k=17}\beta _k D_{i} 1 (t-t_{i}^* = k )}_\text {Post-treatment-effect} + \nu X_{it} + \gamma Z_{it} + c_{i} + \varphi _{t} + \epsilon _{it} \end{aligned}$$

$t_{i}^*$ refers to the quarter of policy implementation, i.e. August 2012 in Eq. 4, *t* the quarter in which the medication is taken for the individual *i*. $1(t-t_{i}^*= k)$ takes a value of 1 when a quarter is $-13,\ldots ,-1,\ldots 17$ relative to the quarter in which the law was implemented, $t_{i}^*$. That is, using the dataset, an observation window of 13 quarters pre-policy change and 17 quarters post-policy change is constructed.

For this analysis, we deviate from the convention of using the reference point as $k=-1$ instead, we use $k=0$. This is because our data are quarterly and the policy was implemented in the middle of a quarter, i.e., August 2012. This timing results in the pre-policy period being within the same quarter (i.e., July 2012), which could blur the distinction between pre and post-policy periods [19]. Additionally, the implementation of the policy, including the dissemination of information, takes time. The time periods in this study are divided into two parts relative to the quarter of policy implementation ($t_{i}^*$), the lead periods (relative periods between $k=0$ to $k=-14$ quarters pre-policy, not including the reference period, $k=0$), labelled as the pre-policy effect, and post-policy quarter (relative periods from the quarter of policy change, $k=1$, to 17 quarters post-policy, $k=17$).

The main coefficients of interest in Eqs. 3 and 4 are $\alpha _k$ and $\beta _k$ and are considered causal effects of the policy on generic medication prescription, where $\alpha _k$ reflects the pre-policy effects and $\beta _k$ post-policy effects on the probability of being prescribed a generic medication in a quarter. $X_{it}$ and $Z_{it}$ are the time-varying covariates for the individuals and the GPs, respectively. $c_i$ captures the individual FE and $\varphi _t$ captures the calendar quarter and year FE. $\epsilon _{it}$ are the usual error terms. The standard errors are clustered at the individual level, since medication naïve is individual specific, and adjusted for heteroskedasticity.

To determine the similarity between the two groups, control and treatment, for relevant individual characteristics, we use two statistical indicators: standardised difference in means and the variance ratio, presented in Table 3. All the observable controls have a near-zero standardised difference in the means and a variance ratio close to one. The results of the covariate balance reveal no selection bias based on individual characteristics of the two groups.

**Table 3:** Independent variables: Balance Between the Treatment and Control Groups

	Treated Group			Control Group			Balance	
Covariates	Mean	Variance	Skewness	Mean	Variance	Skewness	Std-diff	Var-ratio
*Patients Characteristics*								
Female	0.40	0.24	0.41	0.41	0.24	0.35	-0.03	0.99
Charlson Index	1.58	2.28	0.94	1.59	2.77	1.02	-0.01	0.82
No CKD	0.38	0.24	0.48	0.44	0.25	0.22	-0.12	0.96
CKD Stage 1	0.05	0.05	4.17	0.04	0.03	5.06	0.07	1.38
CKD Stage 2	0.16	0.13	1.85	0.13	0.11	2.22	0.09	1.20
CKD Stage 3	0.17	0.14	1.75	0.16	0.14	1.84	0.02	1.05
CKD Stage 4	0.06	0.05	3.84	0.06	0.05	3.85	0.00	1.00
CKD Stage 5	0.02	0.02	7.19	0.02	0.02	7.08	-0.00	0.97
End Stage	0.05	0.05	3.96	0.05	0.05	4.05	0.01	1.04
Undefined	0.11	0.10	2.52	0.10	0.09	2.59	0.01	1.04
Co-payment (%)	9.37	109.22	1.76	9.27	90.59	1.60	0.01	1.21
GP Switching	0.01	0.01	10.06	0.02	0.02	7.86	-0.05	0.62
Specialist Visit	0.73	0.20	-1.02	0.75	0.19	-1.15	-0.05	1.06
*GP Characteristics*								
Age	59.04	20.71	-0.72	58.81	23.02	-0.88	0.05	0.90
Female	0.24	0.18	1.20	0.26	0.19	1.11	-0.03	0.96
# patients (Yearly)	1481.60	81475.37	-1.06	1476.97	82287.80	-1.07	0.02	0.99
GP Density (Pop. 10 million)	6.86	0.90	-0.43	6.86	0.94	-0.48	0.00	0.96
GP Pop. supramed	0.48	0.25	0.08	0.48	0.25	0.06	-0.01	1.00

Notes: The table presents the difference between control and the treatment group. Standardised difference in means and the variance ratio are presented to reveal the balance between the groups. The table presents the mean, variance and skewness of each group and then the standard differences and the variance ratios

## Results

### Regression discontinuity in time

Figure 4 presents the RDiT plot, which shows no evident jump at the implementation of the soft law in quarter 1 2012, suggesting a limited immediate impact of the soft law. Instead, we observe a gradual upward trend in the outcome variable over time, which plateaus starting in 2013. Notably, a greater rate of change appears after quarter 3, 2012, which may reflect the effect of the subsequent hard law. The results from the RDiT models to examine the effect of the soft law are presented in Table 4. Columns (1) and (2) present the estimated LATE with and without the inclusion of covariates. The results indicate that the soft law led to a 0.53 percentage point increase in the probability that a patient received a generic medication at the time of the law’s implementation. Although the result is modest and is not statistically significant at the conventional levels of significance.

**Table 4:** Estimated local average treatment effect of the RDiT

	Soft Law (January 2012)	
	(1)	(2)
Estimate	-0.0033	0.0052
	(0.0042)	(0.0052)
Observations	544,181	583,105
Covariates	Y	N

Notes: The table presents the results from the RDiT model. The outcome variable is the probability of prescription of generic drugs. Columns (1) and (2) present the results for the Soft law, i.e. January 2012, with and without covariates, respectively. Standard errors are clustered at the level of the GP and are presented in parentheses. ***p<0.01, **p<0.05, *p<0.1

### Yearly effects of the 2012 law

**Fig. 1:**
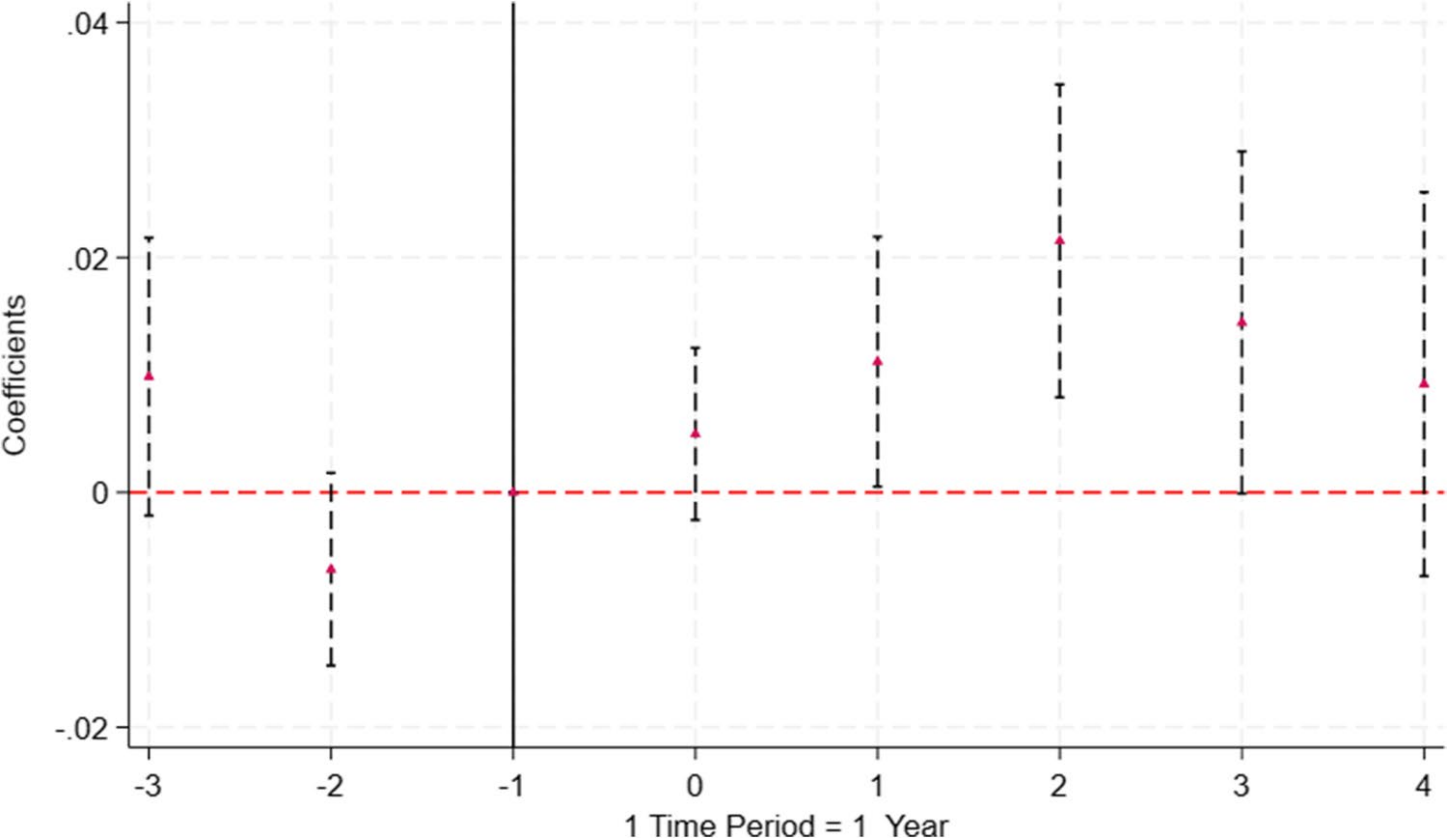
Coefficient Plot for the Effect of the Two Laws on the Prescription of Generic Medications Yearly. Notes: Regression coefficients estimated in Eq. 1 are plotted and the vertical lines indicate the 90% confidence interval. Standard errors are clustered at the individual level. Time periods are calculated with respect to the year of the policy change, 2012 ($t=0$). The year before the policy change is set as the reference year and is shown by the vertical line at $time=-1$. The mean of the outcome variable is 0.40 at $t= -1$

Figure 1 presents the results for the effect of the ’bundled’ policy change in 2012. The results show an increase in probability of prescriptions of generics by 0.05 percentage points in the year of the policy implementation, however, the coefficient is not statistically significant at the conventional levels (1% to 10%).^6^ In the following year, there is a positive, albeit small effect, approximately equal to 1 percentage points (2.5% relative increase from the baseline) relative to one year before the policy change, that is statistically significant at the 5% level. Two years after the implementation of the policy, the results remain statistically significant, with the largest, yet small, increase of approximately 2 percentage points (5% relative increase from the baseline). Four years after, these effects are not longer statistically significant. No anticipation effects are observed, as the coefficients in the pre-period are insignificant.

**Fig. 2:**
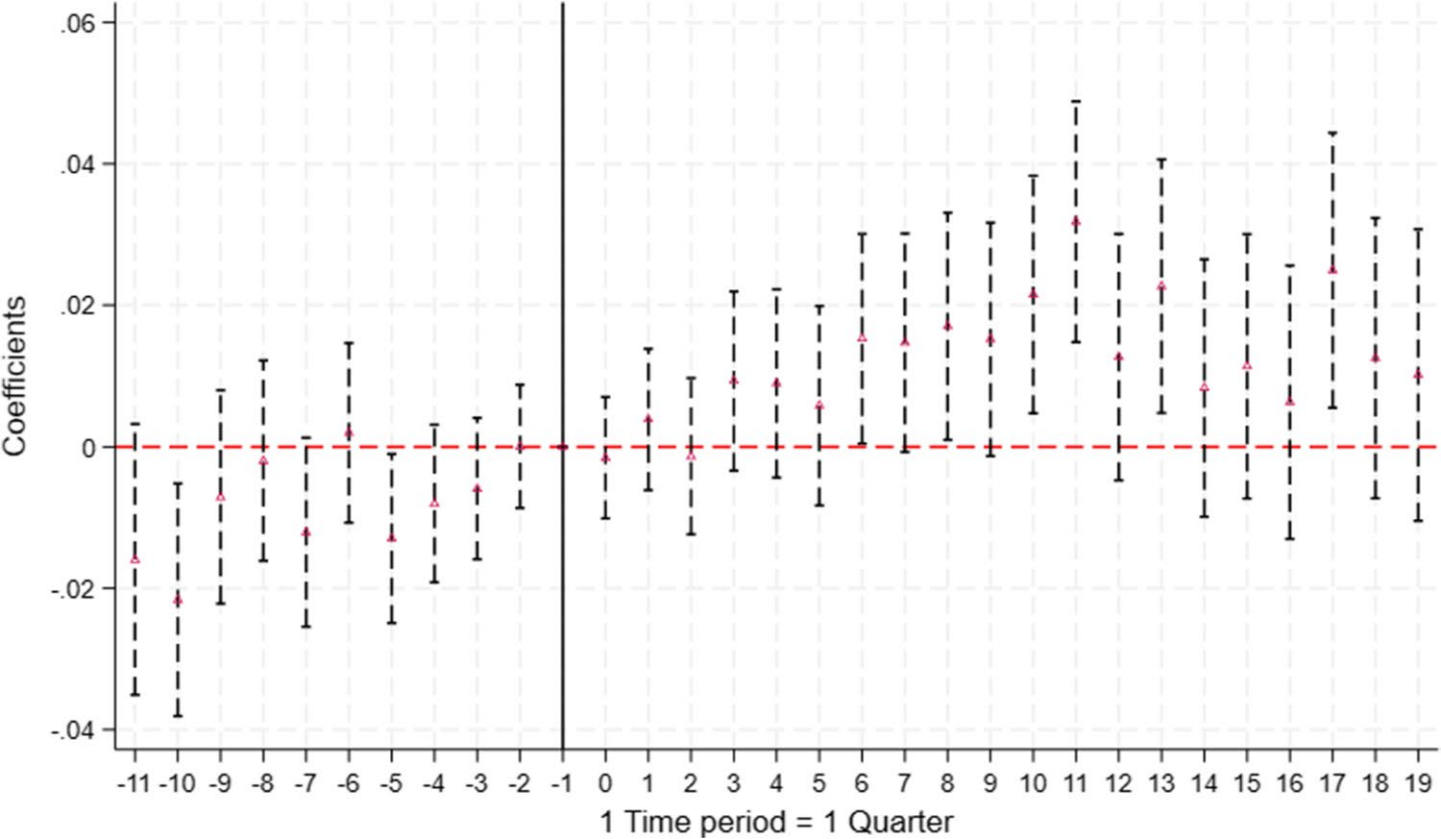
Coefficient Plot for the Effect of the Soft Law (Quarter 13) on the Prescription of Generic Medications. Notes: Regression coefficients estimated in Eq. 2 are plotted and the vertical lines indicate the 90% confidence interval. Standard errors are clustered at the individual level. Time periods are calculated with respect to the quarter of the soft law policy change, January to March 2012 ($t=0$). The quarter before the policy change is set as the reference quarter and is shown by the vertical line at $time=-1$. The mean of the outcome variable is 0.41 at $t= -1$

### Quarterly effects of the soft law

Figure 2, presents the results for the event study that reveals the differences in the probability of prescribing generic medication for naïve patients after the soft law was implemented ($t=0$).^7^ The results show no effect of the soft law in the quarter of implementation and in the following quarter, i.e., in the short run, compared to the reference period. Positive and statistically significant effects at the 10% level are observed from the 6$^\textrm{th}$ quarter after implementation, with an estimated increase of approximately 2 percentage points, relative to a baseline mean of 0.411. These effects remain statistically significant up to the eleventh quarter after implementation, and reappear sporadically in the thirteenth and seventeenth quarters, with magnitudes ranging from approximately 2 to 3 percentage points, representing a relative increase of approximately 5% to 7% over the baseline. This timing suggests that the effects could be driven by the subsequent implementation of the hard law, a potential delay in the enactment of the soft law, or a combination of both factors.

**Fig. 3:**
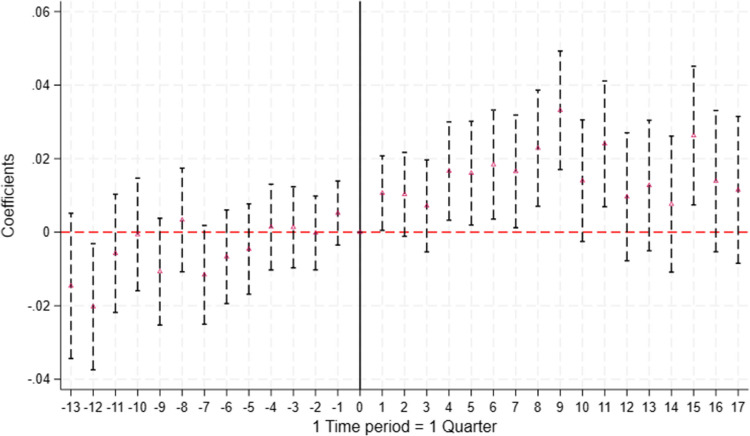
Coefficient Plot for the Effect of the Hard Law (Quarter 15) on the Prescription of Generic Medications. Note: Regression coefficients estimated in Eq. 2 are plotted and the vertical lines indicate the 90% confidence interval. Standard errors are clustered at the individual level. Time periods are calculated with respect to the quarter of the hard law policy change, July to September 2012 ($t=0$). The quarter of the policy change is set as the reference quarter and is shown by the vertical line at $time=0$. The mean of the outcome variable is 0.45 at $t= 0$

### Quarterly effects of the hard law

Figure 3, presents the results for the event study that reveals the differences in the probability of prescribing generic medication for naíve patients in quarters prior and post the hard law was implemented in August 2012 ($t=0$).^8^ The estimates of pre-policy effects ($t=-13$ ...$-1$), i.e., prior to August 2012, are statistically insignificant, while post-policy coefficients ($t =1$ ...17) vary in significance. In particular, positive and significant effects are observed starting at the 4$^\textrm{th}$ quarter (i.e., 1 year after policy change) and remain statistically significance until 9 quarters (2.25 years after policy change) post implementation. These effects are statistically significant at the conventional levels of significance (1% to 10%) with modest magnitudes varying between 1.6 percentage points to 3.2 percentage points. Relative to the baseline mean of 0.45, this corresponds to an increase of approximately 3.6% to 7.1%. These effects are statistically significant in the 11$^\textrm{th}$ and 15$^\textrm{th}$ quarters after the policy change, with estimated increases of 2.4 and 2.6 percentage points, respectively. These represent relative increases of approximately 5.3% and 5.8% over the baseline.

## Robustness checks

To further test the reliability of our estimate, we conduct robustness checks first for the RDIT model and then for the event studies. First, we focus on the potential systematic manipulation of the units near the cut-off point by taking a donut hole approach. It is assumed that in the case of systematic manipulation, observations near the cut-off (Quarter 1, 2012) are likely to be affected, as such observations near the cut-off are excluded. We applied the donut hole approach to our basic model by excluding 1-2 pre-policy quarters. Exclusion of quarters post implementation was avoided due to the implementation of the hard law soon after, risking contamination of the effect. The results presented in Table 7 indicate that the RDiT estimates remain stable overall, however, it is now marginally significant when we exclude one quarter prior.

Next, following the procedures of [9] we use a placebo cut-offs^9^ for falsification analysis for the RDiT estimator. More specifically, we examine if there is any effect on the outcome at various placebo cut-offs. The results presented in Table 8 reveal overall no significant placebo effects.^10^

**Fig. 5:**
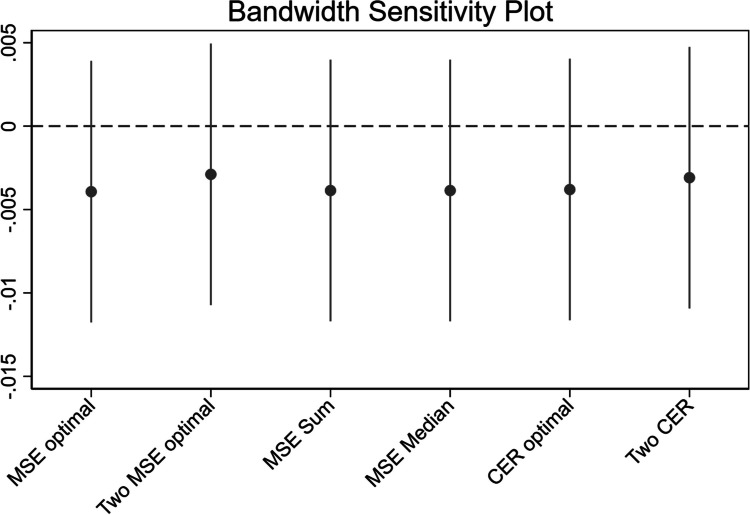
Coefficient plot for RDiT estimate using various bandwidth selection criteria. Notes: The plot presents the coefficient estimates for the RDiT using various bandwidth selectors and the 95% confidence interval

We test for the similarity of the treated units, i.e. all individuals, before and after the cut-off point of the soft law, presented in Table 9. We test the effects of several observable covariates as the outcome variable on the running variable. The results presents no statistically significant results around the cut-off point. However, GP switching, was statistically significant, which is controlled in the regression.^11^ Inclusion or exclusion of units at the end point of the neighbourhood by selecting different bandwidths may affect the RD estimator. Therefore, we check the stability of the estimator against different time bandwidths, which led to similar insignificant estimators, presented in Fig. 5 and Table 10.

**Fig. 6:**
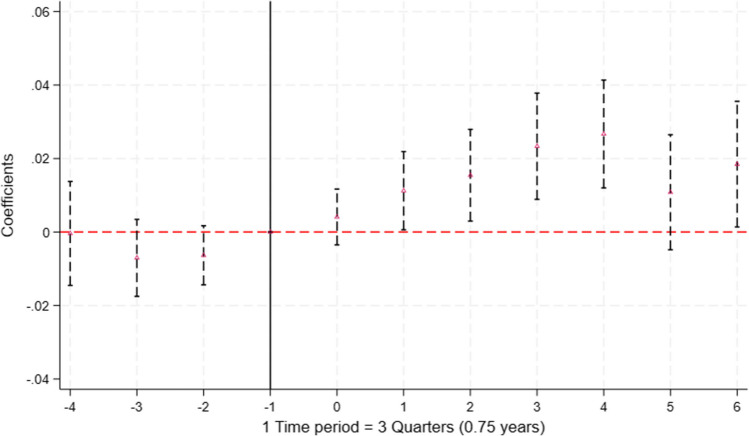
Coefficient Plot for the Effect of the Hard Law on the Prescription of Generic Medications measured in 3 Quarters. Notes: Regression coefficients estimated in Eq. 2. The vertical lines indicate the 90% confidence interval. Standard errors are clustered at the individual level. Time periods are calculated with respect to the first 3 quarter in which the policy (soft and hard law) was enacted, January to September 2012 which is set as a reference period as well and is shown by the vertical line at $time=0$. For convenience, we have omitted the pre-fix of *t* from the x-axis labels

For the event study, since the laws were enacted within a span of 3 quarters (precisely 8 months), we focus on studying the effects in the subsequent 3 quarters (0.75 years) and the following time periods defined in three-quarter intervals (see Fig. 6). This is valuable for policymakers because it offers a more detailed time frame, helping them determine whether the laws were in effect within three quarters, as opposed to the one-year period used in the main analysis. In the analysis, the results show positive and statistically significant effects starting at three quarters after implementation, lasting up to 3 years post-policy change. No such effects are observed in the quarter of implementation.

**Fig. 7:**
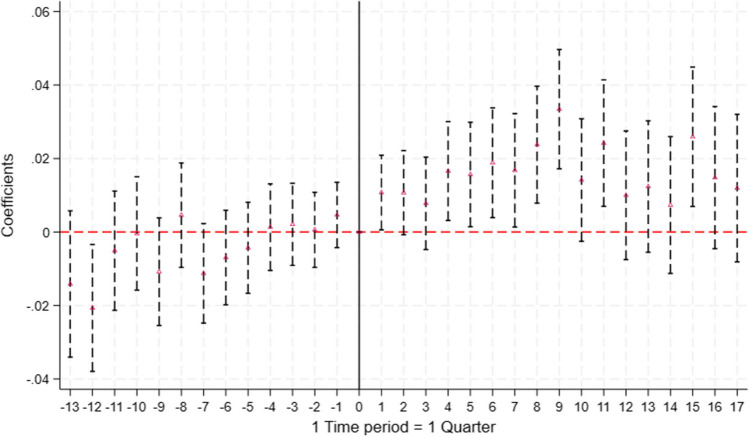
Coefficient Plot for the Effect of the Hard Law (Quarter 15) on the Prescription of Generic Medications Without Individual who Switch GPs. Notes: Regression coefficients estimated in Eq. 2, using a sample of individual who did not switch their GP during the study time period, are plotted. The vertical lines indicate the 90% confidence interval. Standard errors are clustered at the individual level. Time periods are calculated with respect to the quarter of the hard law policy change, July to September 2012. The quarter of the policy change is set as the reference quarter and is shown by the vertical line at $time=0$. For convenience, we have omitted the pre-fix of *t* from the x-axis labels

Next, we excluded individuals who switched their GPs during the study period. This could have occurred for various reasons, such as relocation within the Region, changes in personal preferences, or dissatisfaction with the GP’s promotion of generic medications, which some patients may not prefer. Excluding these individuals ensures that the results are not driven by confounding factors unrelated to the law changes. Figure 7 presents these results, indicating that the hard law had a positive and significant effect one quarter after the policy was implemented, followed by an increase in the probability of the use of generic medication 4 quarters after, which remains statistically significant until 9 quarters after the law was implemented.

**Fig. 8:**
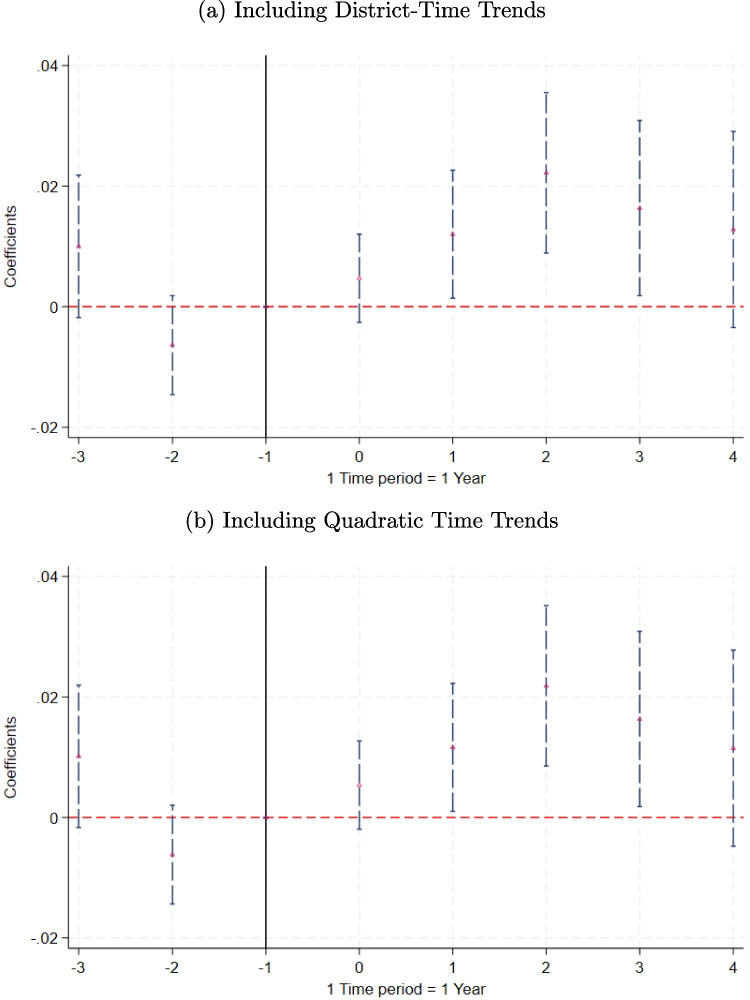
Robustness Checks for the Event Study, Yearly. Notes: Panel (a) and (b) present the regression coefficients estimated in Eq. 1 are plotted and the vertical lines indicate the 90% confidence interval. Panel (a) includes district time trends where Panel (b) includes Quadratic time trend. Standard errors are clustered at the individual level. Time periods are calculated with respect to the year of the policy change, 2012. The year before the policy change is set as the reference year and is shown by the vertical line at $time=-1$. The mean of the outcome variable is 0.40 at $t= -1$

To account for the possibility of non-linear time trends in medication prescribing unrelated to the policy intervention (such as information exposure to the financial budget, availability of new generic drugs), we include quadratic functions of time. The results remain robust to the inclusion of these inclusions, see Fig. 8. We also include interaction terms between time (quarters) and districts, allowing each district to have its own unique trend over time. It could be that not all districts follow the same adoption patterns over time. Using this flexible approach, our results are robust, see Fig. 8.

## Heterogenous effects

Given the tripartite interaction between physicians, patients, and payers, it is important to test for subgroup heterogeneity to assess how supply- and demand-side factors influence prescribing behaviour. To examine this, we investigate two supply-side factors, GP competition and the interaction with specialists, and a demand-side factor, the patient’s income level. We conduct quarterly analyses to estimate these effects with reference to the implementation of the hard law only.

**Fig. 9:**
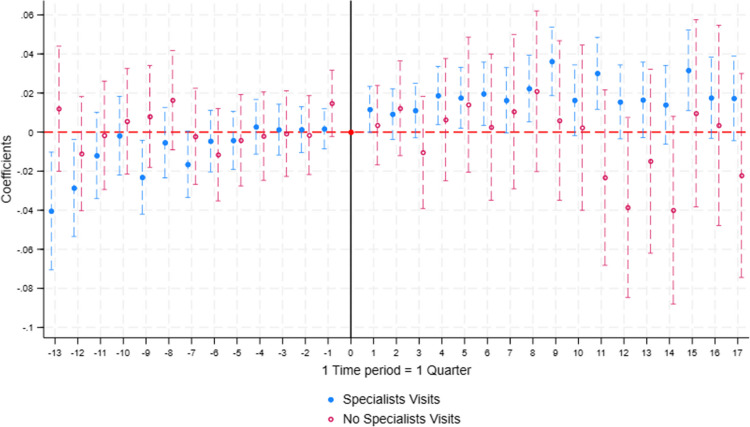
Coefficient Plot for the Effect of the Hard Law (Quarter 15) on the Prescription of Generic Medications by Visits to the Specialist. Notes: The graph presents the coefficient for the dynamic effects of the policy for those who visited the Specialist and those who did not. The vertical lines indicate the 90% confidence interval. The quarter of the policy change is set as the reference quarter and is shown by the vertical line at $time=0$. Relative quarters are calculated with respect to the quarter of the policy change. The y-axis provided the coefficients for each lag and lead. Standard errors are clustered at the individual level. For convenience, we have omitted the pre-fix of *t* from the x-axis labels

The interaction with different types of physicians may influence the prescription of generic medications [18]. CKD patients, like most chronic patients, are likely to visit not only their GPs, but also other specialists in the same quarter or at different quarters. The involvement of specialists could include co-management of patients with GPs, which may shift the main prescribing responsibility to specialists rather than GPs, Pruckner and Schober [32] despite the laws targeting all physicians regardless of their institutional status or affiliation. To understand such effects, we conduct sub-sample analysis for patients who did or did not visit specialists in previous quarters, and hence, were not exposed to the potential influence of the specialists on the prescription choice. For the individuals who interacted with the specialists, we observe from Fig. 9 that the hard law had a positive and statistically significant effect on the probability of taking generic drugs. The results are statistically significant 5 and 6 quarters after the policy change and then in quarters 8, 9, 11 and 15. No similar effects are observed for individuals who had no interactions with the specialists during the observation period.

**Fig. 10:**
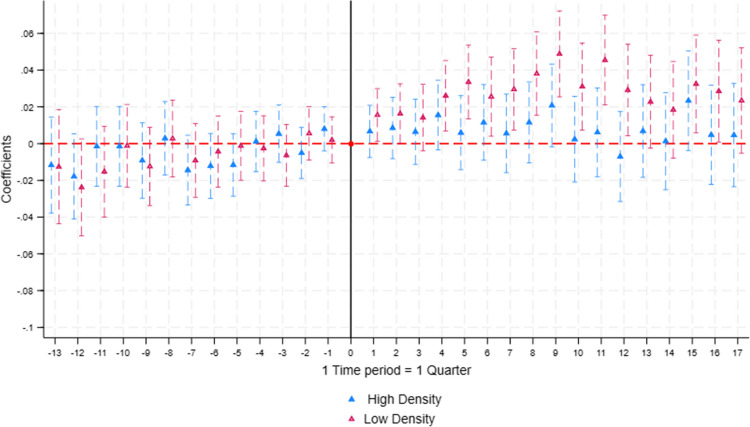
Coefficient Plot for the Effect of the Hard Law (Quarter 15) on the Prescription of Generic Medications by GP Competition. Notes: The graph presents the coefficient for the dynamic effects of the policy for individuals’ GP working in high and low competitive areas. The vertical lines indicate the 90% confidence interval. The quarter of the policy change is set as the reference quarter and is shown by the vertical line at $time=0$. Relative quarters are calculated with respect to the quarter of the policy change. The y-axis provided the coefficients for each lag and lead. Standard errors are clustered at the individual level. For convenience, we have omitted the pre-fix of *t* from the x-axis labels

Competition among GPs can influence the decision to prescribe generic medication [38], as a higher level of competition leads to a greater risk of losing patients and their capitation fees. Consequently, investigating the differential effect of different levels of competition among GPs can reveal important insights. GP density (number of GPs over the population living in a given district) is used to create two subgroups of individuals: those living in districts with a GP density above or below the median level in the Region, indicating a higher (lower) level of competition among GPs. The results presented in Fig. 10 show no effect of the hard law on the probability of prescribing generic medications when GPs practice in competitive areas. On the contrary, when GPs work in low-competitive areas, we observe a statistically significant increase in the probability of prescribing a generic medication in the quarter following the policy implementation. These effects remain significant in the long term, up to 12 quarters after the policy was introduced.

**Fig. 11:**
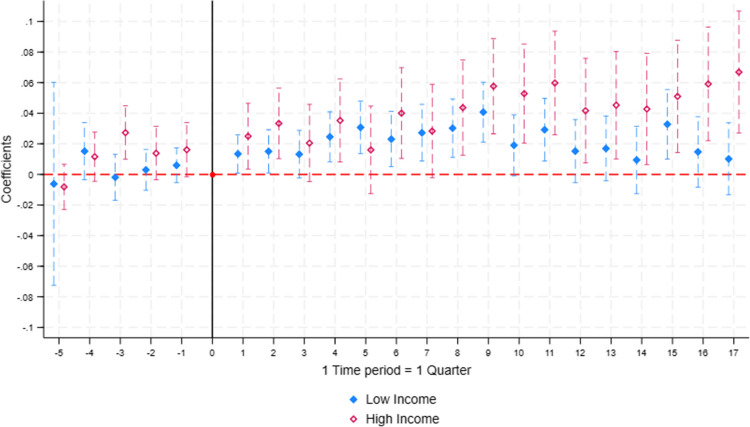
Coefficient Plot for the Effect of the Hard Law (Quarter 15) on the Prescription of Generic Medications by Income. Notes: The graph presents the coefficient for the dynamic effects of the policy for individuals with high and low income. The vertical lines indicate the 90% confidence interval. The quarter of the policy change is set as the reference quarter and is shown by the vertical line at $time=0$. Relative quarters are calculated with respect to the quarter of the policy change. The y-axis provides the coefficients for each lag and lead. Standard errors are clustered at the individual level

On the demand side, although it is widely established that patients perceive branded medications as superior goods to generics ones, demand-side regulations focusing on co-payments of prescription medications could play a role in the decision of patients to demand generic or branded medications [10]. Accordingly, physicians might internalise and consider the patients’ socioeconomic status when making their prescription choice. Information on the socioeconomic characteristics of the patients is used to develop two subsamples to distinguish patients based on the level of income: low-income and non-low-income. The results presented in Fig. 11 show no distinct patterns for both the sub-groups, which are statistically significant and positive in the short and the long run at sporadic intervals.

## Discussion and conclusion

This paper evaluates the effect of two laws that aim to promote generic medications in settings where physicians act as agents to both the patients and the regulators/payers. The institutional setting of the Italian Region Emilia-Romagna provides an opportunity to investigate the effects of two laws with alternative implementation mechanisms: 1) the soft law (January 2012) which required physicians to inform all their patients about the availability of generic medications and 2) the hard law (August 2012) which strictly required physicians to only prescribe generics medication to naïve patients. The results presented show that the soft law had no significant effect on the probability of generic medication prescriptions. The laws bundled together for medication naïve patients, had a positive, yet modest effect on the prescription of generic medications. On the disentanglement of this effect, the hard law seems to have driven this change, while the soft law had no significant effects. These results corroborate similar evidence [16, 27] according to which stricter mandatory laws have greater effects than softer, recommendation based, laws, especially in professional settings where there is room for discretionary choices and monitoring is costly.

Given that the two laws were implemented in close proximity, it is challenging to disentangle their individual effects. Due to the temporal proximity of the hard law, the soft law period might be subject to anticipatory behaviour, potentially confounding the estimate of its isolated impact. It is possible that, in the absence of the hard law, the soft law alone may have been somewhat effective in driving the increase in generic medication prescriptions. This does not invalidate the findings but weakens the clean interpretation of the estimates and calls for cautious causal interpretation. At the same time, the observed effects taking place only after the implementation of the hard law could be the result of the combined influence of recommendations followed by mandates. However, these possibilities seem unlikely, as the soft law itself was not significantly different from previous clinical guidelines.^12^ Additionally, since only modest increases are observed in the prescription of generics, even after the issuance of the hard law, it is more likely that even the mandatory elements of the law were not enforced as effectively as anticipated due to monitoring problems and a lack of significant actions in case of lack of compliance.

The secondary research objectives examined whether the effect of the hard law on the outcome of interest has heterogeneous effects due to both supply and demand side factors. Interaction with specialists, competition among physicians, and the income status of the patients were examined using sub-samples. Our results from the mandatory law had heterogeneous effects across sub-groups. First, naïve patients who interacted with the specialists were more responsive to the mandate to opt for generic medications. This may be due to the fact that specialists mostly work in hospital settings where they are subjected to greater peer-pressure and/or are monitored more closely by the regulator/payer. These results align with those presented in [32] who show that hospital specialists decisions have a significant influence on patients not only while they are hospitalised but also after discharge and with evidence that general practitioners exhibit herding-like behaviour by following specialist’s prescribing choices [45].

Second, for GPs working in less competitive environments, a higher level of compliance with the hard law was observed. No such effect was observed for individuals whose GP works in high competition areas. These findings are consistent with a simple model of physician behaviour, that provides evidence that competition for patients, i.e., high competitive areas, leads physicians to move toward the preferences of marginal patients [12], in our context, preference for branded medications. This is because as the number of physicians increases, each physician caters to fewer patients, ceteris paribus. The demand for a particular physician is more sensitive to the level of service provision, i.e, patients are relatively more demand elastic. Hence physicians are more likely to provide the patients preference of medication, i.e., branded, in high competitive areas. This finding aligns with those of Schaumans [38] and Currie et al. [12], who present that under competitive pressures, prescribing behaviour can be used strategically to retain or attract patients. It could also be that GPs working in less competitive areas are more likely to comply with the guidelines, as there is a low threat of losing patients to other GPs if the patient is not satisfied with the GPs’ decision to prescribe generic medications. Finally, our results did not show great heterogeneity based on income.

One caveat to consider is the presence of digital prescription making electronic records of prescriptions available. This is important because such systems provide access to past prescription data, making GPs’ compliance with guidelines more accountable, as the records can now be verified, unlike paper prescriptions. Additionally, if the system includes pre-filled information favouring generic medications, it may bias the results, particularly if these systems were introduced in close proximity to the 2012 laws. In such a case, the observed effects may be partly driven by the introduction of digital systems rather than the regulations.

In Emilia-Romagna, an electronic prescription system had not yet been initiated at the time of the introduction of the laws. The rollout of the system began only in early 2014. Furthermore, no data are available on the extent of its implementation. While it is possible that the long-run effects observed may be partly attributable to the rollout of this system, our analysis controls for time trends, that capture structural changes across the region. Furthermore, in Italy, there is no predefined or official list of medications required for prescribing and physicians retain substantial discretion. This makes it unlikely that the observed effects are driven by digital prescribing systems rather than by the laws itself.

This research strengthens the existing literature in two ways; first, by analysing two different types of laws, albeit with the same aim and target groups, which offers evidence on the comparative effectiveness of soft law and mandatory regulations. Second, the analysis provides insights into the short, medium, and long-term effects of the two laws through an event study model. Third, we incorporate a unique source of patient-GP linked administrative dataset, with a large sample size, further strengthening the analysis. The limitations include the lack of generality, since this analysis focuses solely on CKD patients, which may not be the case for more severe acute diseases and in cases where only the specialists manages patients. We focus exclusively on the northern region of Emilia-Romagna, with a population of 4.5 million inhabitants. Due to regional disparities within Italy, the findings may not be fully generalisable to all other regions. Finally, the analysis could also be enriched by the inclusion of a comparative control group from areas where the soft law was not implemented.

The findings of this research are relevant for policymakers as they provide evidence on the effectiveness of implementing a soft law and a stricter mandatory law aimed at the objective. The mandatory law is found to be more effective than the soft law for medication naïve patients in increasing the generic drug prescriptions. Even though the results are modest, small increases in generic prescribing can generate substantial system-wide savings, especially if the cost differential with branded drugs is large, which can vary by 20% to 70% [1]. This supports the policy relevance of encouraging even modest shifts in prescribing behaviour, which can be deemed cost-effective, although further research is warranted to quantify the savings at the system level.

An important aspect for policymakers to consider, in this context, is the role of medication naïve patients. At first glance, whether a patient is medication naïve or not should generally not influence the choice between a branded and generic drug. This is because generics are chemically equivalent to their branded counterparts and concerns for adverse consumption outcomes, such as allergies are typically ingredient-specific rather than brand specific. However, individuals who have never been prescribed or have a long washout period may be more price-sensitive and, therefore, more inclined to accept a generic alternative. In contrast, non-naïve patients may be more resistant to a switch due to habit, perceived effectiveness, or concerns about the change. Patients naïvety may also influence GP prescribing behaviour, for instance due to inertia [25, 36], i.e., making GPs more inclined to providing a repeat-prescription for the same medication for non-naïve patients. Such behavioural aspects have important implications for the effectiveness of policies.

Accordingly, more understanding of the reasons behind the lack of effect for the soft law is needed. In addition, the use of monitoring tools and incentive structures for the implementation might be required. Furthermore, understanding the sources of heterogeneity in treatment effects across subgroups enables policymakers to better identify the conditions under which a policy is more likely to be effective. This insight supports the development of tailored strategies, promotional activities, and targeted incentives to enhance the success of a policy. For instance, our findings show that more emphasis is required on the implementation of chronic disease management programs where patients are co-managed by different type of physicians such as GPs or specialist. The effect of the interaction with different physicians’ needs be considered when planning monitoring activities and incentivise tools to support the implementation of similar laws. Similarly, the design of an effective incentive structure should address the different incentives to comply with new regulation by GPs working in areas with different competitive pressures.

## Figures and Tables

**Fig. 4:**
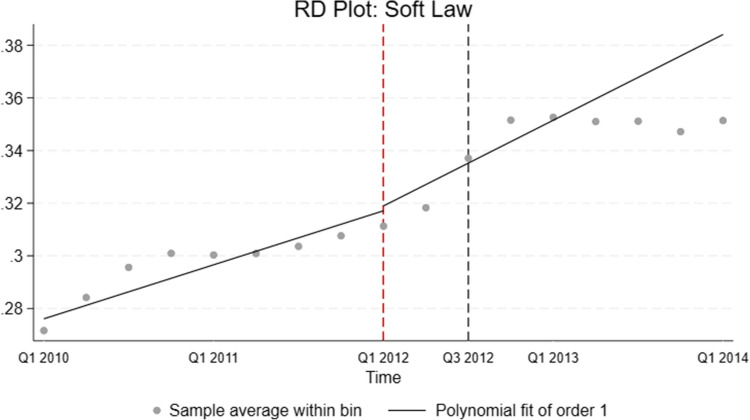
RDiT Plot. Notes: This plot displays the average of generic prescriptions on the vertical axis and time in quarters on the horizontal axis. The soft law was implemented in Quarter 1, 2012, presented with the red dashed line, and the Hard law was implemented in Quarter 3, 2012, presented with the grey dashed line

## Data Availability

Data from the Local Health Authority of Emilia-Romagna are used, which are not permitted to forward or publish. The data are not available upon request.

## Appendix

### A1.2 Event study tables

**Table 5:** Full Regression Results from the Event Study: Yearly Analysis

	Bundled Law
Reference Period	$t-1$
GP variables	
GP Density	-0.001
	(0.003)
GP Switching	0.038***
	(0.010)
GP Female	0.001
	(0.007)
GP Female	-0.008
	(0.009)
GP Age	0.001*
	(0.001)
Patient Variables	
Age	0.019***
	(0.004)
Age Sq.	0.000**
	(0.000)
Charlson Index 1	0.040***
	(0.004)
Charlson Index 2	0.039***
	(0.005)
Charlson Index 3	0.025***
	(0.006)
Charlson Index 4	0.030***
	(0.007)
Charlson Index 5	0.015*
	(0.008)
Charlson Index 6	-0.014
	(0.011)
Charlson Index 7	-0.034**
	(0.016)
Charlson Index 8	-0.054**
	(0.026)
Charlson Index 9	-0.085
	(0.059)
Charlson Index 10	0.072
	(0.119)
Charlson Index 11	0.179***
	(0.053)
CKD Stage 1	0.015*
	(0.008)
CKD Stage 2	0.004
	(0.005)
CKD Stage 3	0.006
	(0.004)
CKD Stage 4	0.002
	(0.006)
CKD Stage 5	-0.006
	(0.010)
End Stage	-0.030***
	(0.008)
Co-payment	-0.008***
	(0.000)
Co-payment lagged	-0.002***
	(0.000)
Medicine Naïve	-0.019*
	(0.010)
Lags and Leads	
$t-3$	0.011
	(0.007)
$t-2$	-0.007
	(0.005)
$t=0$	0.005
	(0.004)
$t+1$	0.011*
	(0.006)
$t+2$	0.021***
	(0.008)
$t+3$	0.014
	(0.009)
$t+4$	0.009
	(0.010)
Constant	-1.442***
	(0.153)
Observations	237,370
Number of ID	42,660
R-squared	0.066

Notes: Regression coefficients estimated in Eq. 2 are presented in this Table. Standard errors are clustered at the individual level. ID represents the number of individuals. Time periods are calculated with respect to the year of laws. Time and district fixed effect are included. ***p<0.01, **p<0.05, *p<0.1

**Table 6:** Full Regression Results from the Event Study: Quarterly Analysis

	(1)	(2)
	Soft Law	Hard Law
Reference Period	$t-1$	$t+0$
GP variables		
GP Density	7.47e-05	7.47e-05
	(0.003)	(0.003)
GP Switching	0.0172***	0.0172***
	(0.004)	(0.004)
Female	0.00342	0.00342
	(0.007)	(0.007)
Group Practice	-0.00282	-0.00282
	(0.008)	(0.008)
Age	0.000873*	0.000873*
	(0.000)	(0.000)
Patient Variables		
Age	0.0229***	0.0229***
	(0.004)	(0.004)
Age Sq.	5.97e-05**	5.97e-05**
	(0.000)	(0.000)
Charlson Index 1	0.0406***	0.0406***
	(0.004)	(0.004)
Charlson Index 2	0.0419***	0.0419***
	(0.004)	(0.004)
Charlson Index 3	0.0320***	0.0320***
	(0.005)	(0.005)
Charlson Index 4	0.0342***	0.0342***
	(0.006)	(0.006)
Charlson Index 5	0.0255***	0.0255***
	(0.008)	(0.008)
Charlson Index 6	0.00615	0.00615
	(0.010)	(0.010)
Charlson Index 7	-0.0106	-0.0106
	(0.014)	(0.014)
Charlson Index 8	-0.0255	-0.0255
	(0.023)	(0.023)
Charlson Index 9	-0.0438	-0.0438
	(0.050)	(0.050)
Charlson Index 10	-0.0622	-0.0622
	(0.097)	(0.097)
Charlson Index 11	0.170***	0.170***
	(0.051)	(0.051)
CKD Stage 1	0.0198***	0.0198***
	(0.007)	(0.007)
CKD Stage 2	0.0119***	0.0119***
	(0.004)	(0.004)
CKD Stage 3	0.0155***	0.0155***
	(0.004)	(0.004)
CKD Stage 4	0.0155***	0.0155***
	(0.005)	(0.005)
CKD Stage 5	0.0127	0.0127
	(0.008)	(0.008)
End Stage	-0.00193	-0.00193
	(0.007)	(0.007)
Undefined	0.0189***	0.0189***
	(0.004)	(0.004)
Co-payment (%)	-0.00789***	-0.00789***
	(0.000)	(0.000)
Lagged Co-payment	-0.00191***	-0.00191***
	(0.000)	(0.000)
Medicine Naïve	-0.0159	-0.0170*
	(0.010)	(0.009)
Lags and Leads		
$t-13$		-0.0152
		(0.012)
$t-12$		-0.0208**
		(0.010)
$t-11$	-0.0164	-0.00642
	(0.012)	(0.010)
$t-10$	-0.0220**	-0.00140
	(0.010)	(0.009)
$t-9$	-0.00759	-0.0116
	(0.009)	(0.009)
$t-8$	-0.00257	0.00256
	(0.009)	(0.009)
$t-7$	-0.0127	-0.0123
	(0.008)	(0.008)
$t-6$	0.00140	-0.00734
	(0.008)	(0.008)
$t-5$	-0.0135*	-0.00512
	(0.007)	(0.007)
$t-4$	-0.00851	0.00106
	(0.007)	(0.007)
$t-3$	-0.00629	0.00117
	(0.006)	(0.007)
$t-2$	-0.000102	-0.000361
	(0.005)	(0.006)
$t-1$		0.00519
		(0.005)
$t=0$	-0.00153	
	(0.005)	
$t+1$	0.00402	0.0108*
	(0.006)	(0.006)
$t+2$	-0.00117	0.0103
	(0.007)	(0.007)
$t+3$	0.00960	0.00709
	(0.008)	(0.008)
$t+4$	0.00909	0.0166**
	(0.008)	(0.008)
$t+5$	0.00593	0.0159*
	(0.009)	(0.009)
$t+6$	0.0155*	0.0180**
	(0.009)	(0.009)
$t+7$	0.0148	0.0163*
	(0.009)	(0.009)
$t+8$	0.0168*	0.0226**
	(0.010)	(0.010)
$t+9$	0.0151	0.0329***
	(0.010)	(0.010)
$t+10$	0.0215**	0.0137
	(0.010)	(0.010)
$t+11$	0.0317***	0.0235**
	(0.010)	(0.010)
$t+12$	0.0125	0.00909
	(0.011)	(0.011)
$t+13$	0.0223**	0.0120
	(0.011)	(0.011)
$t+14$	0.00792	0.00696
	(0.011)	(0.011)
$t+15$	0.0109	0.0256**
	(0.011)	(0.011)
$t+16$	0.00579	0.0133
	(0.012)	(0.012)
$t+17$	0.0245**	0.0108
	(0.012)	(0.012)
$t+18$	0.0122	
	(0.012)	
$t+19$	0.00968	
	(0.013)	
Constant	-1.745***	-1.745***
	(0.162)	(0.162)
Observations	805,621	805,621
R-squared	0.052	0.052
Number of ID	42,447	42,447

Notes: Regression coefficients estimated in Eqs. 3 and 4 are presented in this Table. Standard errors are clustered at the individual level. ID represents the number of individuals. Time periods are calculated with respect to the quarter of law implemented for the hard law and the quarter prior to the implementation of the soft law. Time and district fixed effect are included. ***p<0.01, **p<0.05, *p<0.1

### A1.3 Robustness checks

#### RDiT donut hole approach

**Table 7:** Donut Hole Approach

Excluding Quarters before	1 (Quarter 4, 2011)	2 (Quarter 3, 2011)
RD Estimate	-0.009**	0.002
	(0.004)	(0.004)
Observations	502,025	502,801

Notes: Each column represents a separate RDiT regression estimate. Each column corresponds to results from different model specifications, first excluding quarter 4, 2011 and excluding quarter 3, 201. Standard errors are reported in parentheses. ^*^$p<0.1$, ^**^$p<0.05$, ^***^$p<0.01$

#### RDiT placebo

**Table 8:** RDiT Placebo Estimates for Different Quarter Cutoffs

Column	(1)	(2)	(3)	(4)	(5)	(6)	(7)
Year	2011	2011	2012	2012	2013	2013	2013
Quarter	Q3	Q4	Q2	Q4	Q1	Q2	Q3
RD Estimate	0.000	-0.002	0.005	-0.003	-0.015***	-0.006	0.001
	(0.004)	(0.004)	(0.004)	(0.005)	(0.006)	(0.005)	(0.005)
Observations	572,175	572,175	572,175	572,175	572,175	572,175	572,175

Notes: The table presents the results from the RDiT model. The outcome variable is the probability of prescribing generic drugs. Each column corresponds to results from different model specifications, and the subsequent rows indicate the year and quarters used as a placebo. Standard errors are in parentheses. *** p<0.01, ** p<0.05, * p<0.1

#### Local average treatment effect of predetermined covariates

**Table 9:** Estimated Local Average Treatment Effect of Predetermined Covariates

	Patients			GP			
	Age	Gender	CKD Stage	Age	Gender	Switched	Group
RD Estimate	0.78***	0.00	37.43	0.003	0.000	0.006**	0.001
	(0.099)	(0.003)	(25.74)	(0.13)	(0.01)	(0.003)	(0.01)
Observations	583,105	583,105	583,105	583,105	583,105	583,105	583,105

Notes: Each column represents a separate RDiT regression estimate. Standard errors are reported in parentheses. ^*^$p<0.1$, ^**^$p<0.05$, ^***^$p<0.01$

#### Local average treatment effect using different bandwidths

**Table 10:** Estimated local average treatment effect of the RDiT for different bandwidths

	MSE-optimal	Two MSE	MSE Sum	MSE Median	CER-optimal	Two CER
Coefficient	-0.004	-0.003	-0.004	-0.004	-0.004	-0.003
	(0.004)	(0.004)	(0.004)	(0.004)	(0.004)	(0.004)
Observations	533,737	533,738	533,739	533,740	533,741	533,742

Notes: The table presents the different bandwidth selectors used to choose the bandwidth. MSE stands for Mean Squared Error, and CER stands for Coverage Error Rate. These bandwidth selectors are based on the works of [9]. Standard errors are reported in parentheses

### A1.4 Heterogenous effects

**Table 11:** Heterogenous Effects of the Hard Law: Event Study

	(1)	(2)	(3)	(4)	(7)	(8)
	Specialists Visits	No Specialists Visits	GP Density High	GP Density Low	Low Income	High Income
$t-13$	-0.0406**	0.0120	-0.0113	-0.0127		-0.0234
	(0.018)	(0.019)	(0.016)	(0.019)		(0.024)
$t-12$	-0.0286*	-0.0111	-0.0176	-0.0239		-0.0216
	(0.015)	(0.018)	(0.014)	(0.016)		(0.021)
$t-11$	-0.0120	-0.00167	-0.00121	-0.0153		-0.0231
	(0.013)	(0.017)	(0.013)	(0.015)		(0.019)
$t-10$	-0.00163	0.00554	-0.00111	-0.00108		-0.00252
	(0.012)	(0.016)	(0.013)	(0.014)		(0.018)
$t-9$	-0.0229**	0.00800	-0.00883	-0.0123		-0.0197
	(0.011)	(0.016)	(0.012)	(0.013)		(0.017)
$t-8$	-0.00511	0.0164	0.00331	0.00281		-0.00923
	(0.011)	(0.015)	(0.012)	(0.013)		(0.017)
$t-7$	-0.0163	-0.00222	-0.0140	-0.00912		-0.00849
	(0.010)	(0.015)	(0.012)	(0.012)		(0.016)
$t-6$	-0.00442	-0.0116	-0.0117	-0.00427	-0.0387	-0.00813
	(0.010)	(0.014)	(0.011)	(0.012)	(0.067)	(0.015)
$t-5$	-0.00409	-0.00417	-0.0111	-0.00124	-0.00804	-0.0182
	(0.009)	(0.014)	(0.010)	(0.011)	(0.042)	(0.014)
$t-4$	0.00281	-0.00206	0.00137	-0.00256	0.0149	0.00218
	(0.008)	(0.014)	(0.010)	(0.011)	(0.011)	(0.013)
$t-3$	0.00134	-0.000820	0.00559	-0.00643	-0.00227	0.0184
	(0.008)	(0.013)	(0.009)	(0.010)	(0.009)	(0.011)
$t-2$	0.00140	-0.00158	-0.00498	0.00555	0.00261	0.00520
	(0.007)	(0.012)	(0.008)	(0.009)	(0.008)	(0.010)
$t-1$	0.00172	0.0147	0.00815	0.00196	0.00555	0.00771
	(0.006)	(0.010)	(0.007)	(0.008)	(0.007)	(0.009)
$t+1$	0.0117	0.00351	0.00683	0.0156*	0.0130*	0.0167
	(0.007)	(0.012)	(0.009)	(0.009)	(0.008)	(0.011)
$t+2$	0.00922	0.0122	0.00865	0.0162	0.0147*	0.0250**
	(0.008)	(0.015)	(0.010)	(0.010)	(0.009)	(0.012)
$t+3$	0.0111	-0.0104	0.00661	0.0141	0.0128	0.0122
	(0.009)	(0.017)	(0.011)	(0.011)	(0.009)	(0.013)
$t+4$	0.0186**	0.00635	0.0155	0.0260**	0.0241**	0.0268*
	(0.009)	(0.019)	(0.012)	(0.012)	(0.010)	(0.015)
$t+5$	0.0176*	0.0140	0.00599	0.0334***	0.0303***	0.00749
	(0.009)	(0.021)	(0.012)	(0.012)	(0.010)	(0.016)
$t+6$	0.0196**	0.00249	0.0117	0.0254*	0.0226**	0.0315*
	(0.010)	(0.023)	(0.013)	(0.013)	(0.011)	(0.016)
$t+7$	0.0163	0.0105	0.00581	0.0294**	0.0268**	0.0199
	(0.010)	(0.024)	(0.013)	(0.013)	(0.011)	(0.017)
$t+8$	0.0223**	0.0209	0.0118	0.0380***	0.0298**	0.0351**
	(0.010)	(0.025)	(0.013)	(0.014)	(0.012)	(0.017)
$t+9$	0.0362***	0.00591	0.0211	0.0487***	0.0403***	0.0491***
	(0.011)	(0.025)	(0.014)	(0.014)	(0.012)	(0.017)
$t+10$	0.0163	0.00227	0.00254	0.0310**	0.0186	0.0443**
	(0.011)	(0.026)	(0.014)	(0.014)	(0.012)	(0.018)
$t+11$	0.0301***	-0.0232	0.00635	0.0454***	0.0288**	0.0512***
	(0.011)	(0.027)	(0.015)	(0.015)	(0.012)	(0.019)
$t+12$	0.0155	-0.0386	-0.00686	0.0291*	0.0148	0.0331*
	(0.012)	(0.028)	(0.015)	(0.015)	(0.013)	(0.020)
$t+13$	0.0165	-0.0149	0.00705	0.0227	0.0165	0.0367*
	(0.012)	(0.029)	(0.015)	(0.015)	(0.013)	(0.020)
$t+14$	0.0140	-0.0400	0.00151	0.0184	0.00898	0.0343
	(0.012)	(0.029)	(0.016)	(0.016)	(0.013)	(0.021)
$t+15$	0.0316**	0.00964	0.0234	0.0325**	0.0323**	0.0425**
	(0.013)	(0.029)	(0.016)	(0.016)	(0.014)	(0.021)
$t+16$	0.0176	0.00347	0.00491	0.0285*	0.0142	0.0507**
	(0.013)	(0.031)	(0.016)	(0.017)	(0.014)	(0.022)
$t+17$	0.0173	-0.0222	0.00476	0.0234	0.00965	0.0584**
	(0.013)	(0.032)	(0.017)	(0.017)	(0.014)	(0.023)
Constant	0.642*	-2.939**	0.375	0.453	0.0238	0.688
	(0.368)	(1.358)	(0.490)	(0.516)	(0.467)	(0.632)
Observations	600,642	204,979	432,925	372,696	314,446	301,923
Number of ID	34,899	23,003	28,811	25,094	25,909	26,618

Notes: The Table reports event study estimates from Eq. 1. Periods after $t=0$ include relative quarters after law implementation. The coefficient of the lags and the leads capture the probability of prescribing generic medication over and above that in the reference period, $t=0$. All regressions include Area, Quarter FE. Standard errors are clustered at the individual level. ***p<0.01, **p<0.05, *p<0.1

## Footnotes

1 In Italy, the consumption of generic medicines remains low, especially when compared to other European countries [24]. In 2023, in Italy total pharmaceutical expenditure was 36.2 billion Euros, of which 68.7% was reimbursed by the NHS. Generic medications account for 23% of this expenditure, and 31% in terms of consumption.

2 Due to data limitations, we only have such rich administrative data for those with CKD.

3 The duration of the washout period is not clear in the literature. It is usually case based and depends on the judgment of the clinician [5].

4 A payment method in which a health care provider receives a set amount of payment in advance to cover the predicted cost of a patient’s health care services over a specific period of time.

5 In our data, we are unable to measure patient compliance, while this is an important area for future research, it also falls outside the scope of the current paper.

6 Since we have Quarterly data, we take the average of the generic medication indicator over the quarters the individual is observed in a year. See Table 5 for the regression results.

7 See Table 6 for the regression results.

8 See Table 6 for the regression results.

9 In RDiT designs, “placebo cut-offs” refer to fake or artificial policy implementation dates or quarters used as falsification robustness checks to test whether the law drives observed effects or are artifacts of underlying time trends. This usage is distinct from the medical definition of “placebo” as an inactive treatment in randomised trials.

10 We find a small but significant effect using Quarter 1 2013 as a placebo cut-off, the latter could be capturing the effect of the subsequent hard law.

11 Age of the patient is also significant however this is because time is the running variable, although we control for for age-related risk covariates such as the intensity of the CKD stage and the existence of multi-morbidity; both covariates yielded insignificant effects.

12 Robustness checks using placebo cut-offs also present an insignificant effect in Quarter 1, 2012 and Quarter 2, 2012, coupled with the enactment of the hard mandate in Quarter 3, 2012, is suggestive of the inefficacy of the soft mandate.
